# Identification of One *O*-Methyltransferase Gene Involved in Methylated Flavonoid Biosynthesis Related to the UV-B Irradiation Response in *Euphorbia lathyris*

**DOI:** 10.3390/ijms25020782

**Published:** 2024-01-08

**Authors:** Wanli Zhao, Long Huang, Shu Xu, Junzhi Wu, Fan Wang, Pirui Li, Linwei Li, Mei Tian, Xu Feng, Yu Chen

**Affiliations:** Jiangsu Key Laboratory for the Research and Utilization of Plant Resources, Jiangsu Province Engineering Research Center of Eco-Cultivation and High-Value Utilization of Chinese Medicinal Materials, Institute of Botany, Jiangsu Province and Chinese Academy of Sciences (Nanjing Botanical Garden, Mem. Sun Yat-Sen), Nanjing 210014, China; zhaowanlitcm@126.com (W.Z.);

**Keywords:** *Euphorbia lathyris*, UV-B irradiation, methyltransferase

## Abstract

Flavonoids are ubiquitous polyphenolic compounds that play a vital role in plants’ defense response and medicinal efficacy. UV-B radiation is a vital environmental regulator governing flavonoid biosynthesis in plants. Many plants rapidly biosynthesize flavonoids as a response to UV-B stress conditions. Here, we investigated the effects of flavonoid biosynthesis via UV-B irradiation in *Euphorbia lathyris*. We found that exposure of the *E. lathyris* callus to UV-B radiation sharply increased the level of one *O*-methyltransferase (*ElOMT1*) transcript and led to the biosynthesis of several methylated flavonoids. The methyltransferase ElOMT1 was expressed heterologously in *E. coli*, and we tested the catalytic activity of recombinant *ElOMT1* with possible substrates, including caffeic acid, baicalin, and luteolin, in vitro. ElOMT1 could efficiently methylate when the hydroxyl groups were contained in the core nucleus of the flavonoid. This molecular characterization identifies a methyltransferase responsible for the chemical modification of the core flavonoid structure through methylation and helps reveal the mechanism of methylated flavonoid biosynthesis in Euphorbiaceae. This study identifies the *O*-methyltransferase that responds to UV-B irradiation and helps shed light on the mechanism of flavonoid biosynthesis in *Euphorbia lathyris*.

## 1. Introduction

Plants have evolved various strategies to overcome the detrimental effects resulting from UV-B radiation [1]. One defense strategy is the production of additional antioxidant protectants, such as flavonoids [2,3]. Flavonoid biosynthesis and accumulation are both developmentally and environmentally controlled in plants. UV-B radiation is an environmental stimulus that regulates flavonoid biosynthesis at the transcriptional level of the gene that encodes the corresponding enzymes of the biosynthetic pathway [4]. Flavonoids are well-known natural products with a wide range of biological activities that are beneficial for human health, and they have been used in treating and preventing many diseases [5]. It has been demonstrated that methylated flavonoids have received more attention due to the potential of methylation to alter their biochemical properties and consequently enhance their medicinal effects [6,7,8].

The process of methylation is catalyzed using a methyltransferase that transfers a methyl group from *S*-adenosyl-L-methionine to an appropriate substrate. *O*-methylation occurs as one vital step in many plants’ secondary metabolic biosynthesis, which involves flavonoids [9,10], terpenoids [11,12], and alkaloids [13,14]. *O*-methyltransferase is responsible for the formation of the structural diversity of flavonoids through methylation in plants. Therefore, the identification of *O*-methyltransferases helps us understand the biosynthesis of flavonoids in plants and their role in abiotic stress. Recently, the *O*-methyltransferase genes of flavonoid biosynthesis have been identified in many medicinal plants such as *Isatis indigotica* [15], *Scutellaria baicalensis* [9], and *Pueraria lobate* [16]. However, there are few reports on the involvement of methyltransferases in the plant response to abiotic stress.

*Euphorbia lathyris* L. originated from Europe and is now wild or cultivated, being distributed in multiple regions around the world. *E. lathyris* is known for its great value in medicinal development [17]. Phytochemical research has indicated that the main chemical composition of the medicinal effects of *E. lathyris* comprises diterpenoids and flavonoids [18]. However, the processes of methylation involved in flavonoid biosynthesis remain unknown in *E. lathyris*. Herein, we performed transcriptomic analysis to reveal the biosynthetic candidate genes of methylated flavonoids in *E. lathyris*. We screened one *O*-methyltransferase gene, ElOMT, that is involved in the response to UV induction. *ElOMT1* was expressed in *E. coli*, and we tested its catalytic activities with several possible substrates in vitro. It could be one of the potential genes for agricultural applications. Our findings provide a potential gene for coping with UV-B stress in plants and lay a foundation for the biosynthesis of methylated flavonoids through metabolic engineering.

## 2. Results and Discussion

### 2.1. RNA Sequence Analysis

**Figure 1:**
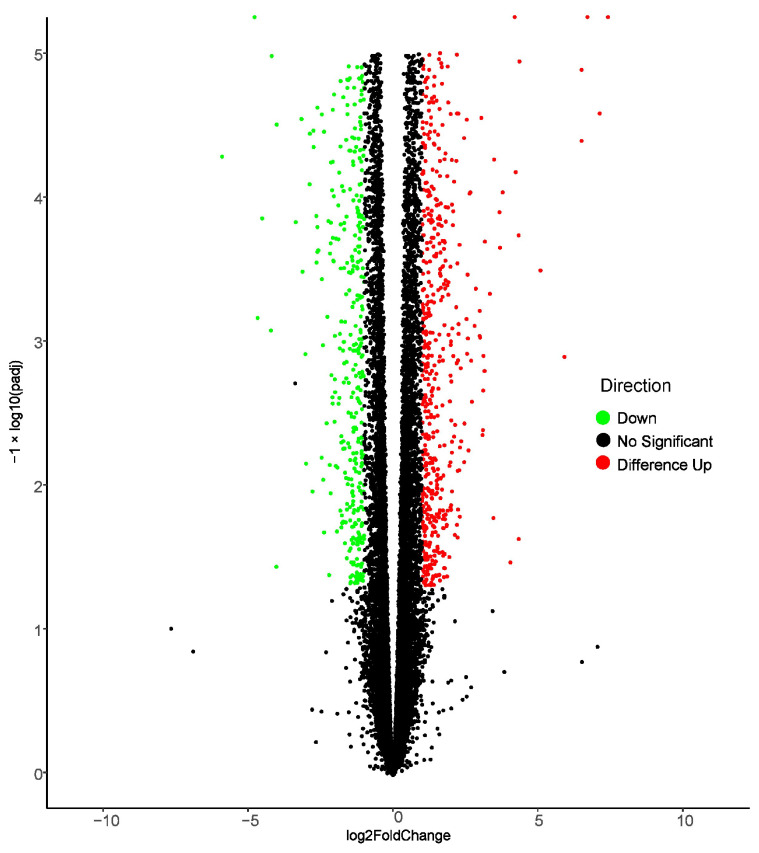
Volcano map of differentially expressed genes after UV-B irradiation of *E. lathyris* callus (ELC-UV0h vs. ELC-UV3h). ELC-UV0h: the callus of *E. lathyris* was not exposed to UV-B irradiation; ELC-UV3h: the callus of *E. lathyris* was exposed to UV-B irradiation for 3 h. FoldChange: the differential fold change in gene expression.

**Figure 2:**
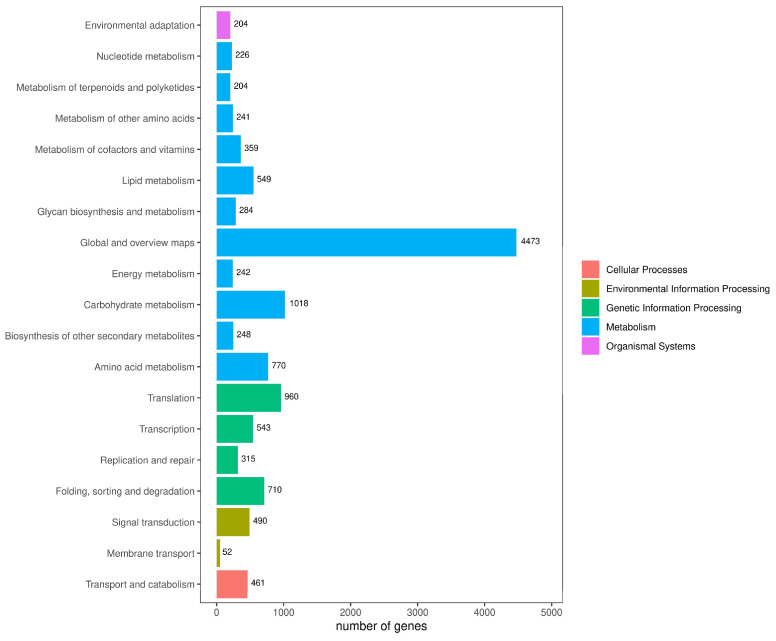
KEGG metabolic pathway classification of *E. lathyris*.

Our preliminary experimental results showed that the flavonoid pathway-related genes were enhanced in *E. lathyris* callus. Therefore, we selected *E. lathyris* callus as the experimental material for UV-B treatment. To analyze the effect of UV-B irradiation on the transcription level of an *E. lathyris* callus, transcriptome sequencing was performed on the *E. lathyris* callus before and after UV-B irradiation, and the results were aligned to the reference transcripts of the *E. lathyris* genome. The results demonstrated a total of 39.50–57.88 million raw reads and 39.34–57.64 million clean reads from the six samples that were obtained. The Q20 and Q30 bases were greater or equal to 97.31% and 92.48% (Table S1). Differential gene expression analysis was performed using read count data from the expression-quantified transcription results of the data analysis. Making comparisons between the two groups, the control group ELC-UV0h (no UV-B treatment) vs. the experimental group ELC-UV3h (UV-B irradiation for 3 h) showed 2095 differentially expressed genes (up, 1549; down, 1356; Figure 1). After the Kyoto Encyclopedia of Genes and Genomes (KEGG) annotation of the transcript sequences, they were classified into several categories, including environmental information processing, genetic information processing, organismal systems, cellular processes, and metabolism according to the KEGG metabolic pathways. Furthermore, 204 genes were involved in the biosynthesis of other secondary metabolites (Figure 2).

### 2.2. Phylogeny of MlOMT Candidates

**Figure 3:**
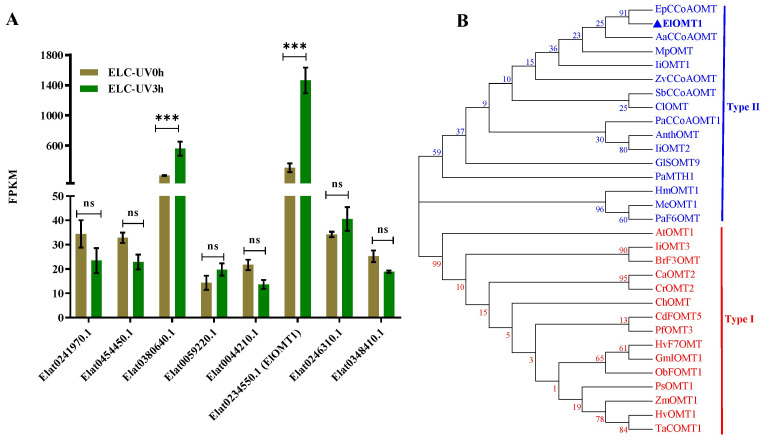
Relative expression levels and phylogenetic tree of methyltransferase genes. Relative expression levels of ElOMT genes measured through FPKM (**A**). The genes with low expression (FPKM less than 20) in different samples are not listed. Bars represent the mean ± SD of three biological replicates. The Student’s t-test was used to analyze gene expression data. The level of significance was set at *** *p* < 0.001. The phylogenetic tree of candidate ElOMT1 and previously characterized plant methyltransferases was constructed using the maximum likelihood (ML) with 1000 bootstrap replicates (**B**). GenBank accession numbers and sequence of *O*-methyltransferase proteins in this tree are listed in Table S2 and Supporting Information S2.

We focused on the methyltransferase gene of *E. lathyris*, and the callus was compared before and after UV treatment. After UV-B irradiation, the expression levels of several methyltransferase genes were significantly changed. Among them, the fold change of *ELOMT1* was the highest, which increased 4.7 times (Figure 3A). For further insights into the potential functions of ElOMT1, we conducted a phylogenetic analysis of the ElOMT1 protein sequences with methyltransferases from other plants that were previously characterized. The analysis divided these OMTs into two subfamilies (Type I: red and Type II: blue; Figure 3B). ElOMT1 belonged to the caffeoyl-CoA *O*-methyltransferase (Type II) and was clustered with EpCCoAOMT from *Euphorbia peplus*. This suggests that ElOMT1 might have the caffeoyl-CoA *O*-methyltransferase function, which catalyzes flavonoid methylation.

### 2.3. In Vitro Characterization of Recombinant ElOMT1

**Figure 4:**
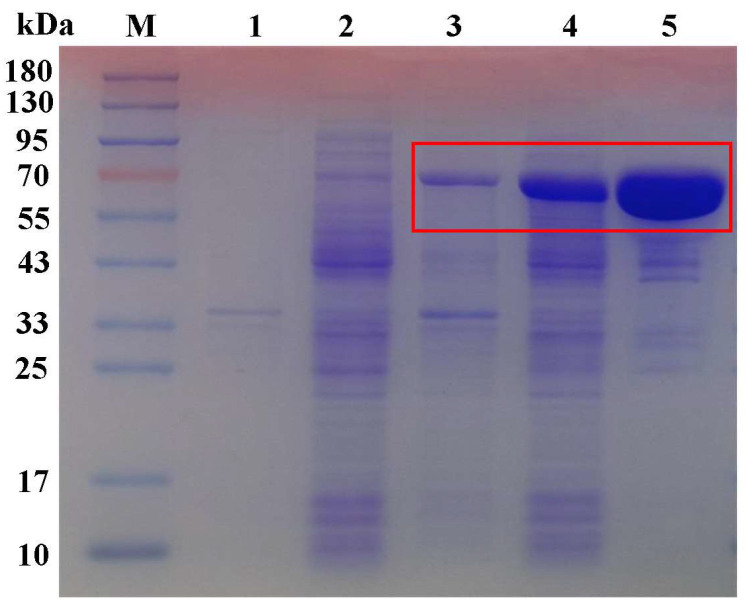
Results of SDS-PAGE of ElOMT1. M: marker; lane 1: pellet of pMAL-c4x (empty vector); lane 2: supernatant of pMAL-c4x; lane 3: pellet of ElOMT1; lane 4: supernatant of ElOMT1; lane 5: purified ElOMT1 (MBP Tag ≈ 42 KDa). Red box: the band of recombinant protein ElOMT1.

To detect the expression of recombinant ElOMT1, the protein was detected using sodium dodecyl sulfate–polyacrylamide gel electrophoresis (SDS–PAGE), which showed specific bands (Figure 4). The induced protein size was consistent with the theoretical prediction of the size of ElOMT1.

**Figure 5:**
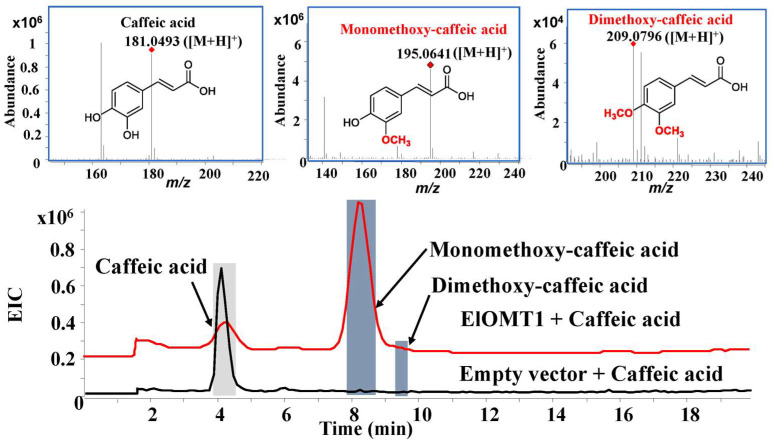
Results of caffeic acid substrate catalyzed by ElOMT1 in vitro. EIC, extract ion chromatogram.

**Figure 6:**
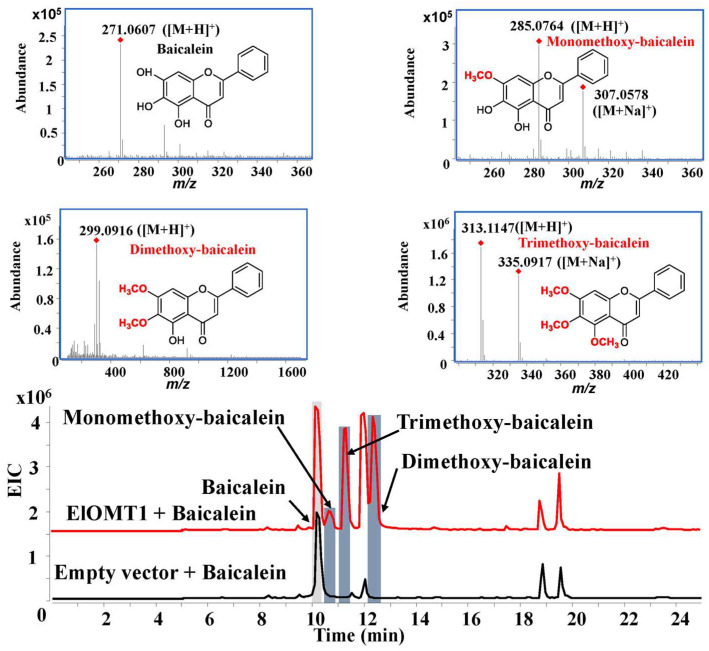
Results of baicalein substrate catalyzed by ElOMT1 in vitro. EIC, extract ion chromatogram.

**Figure 7:**
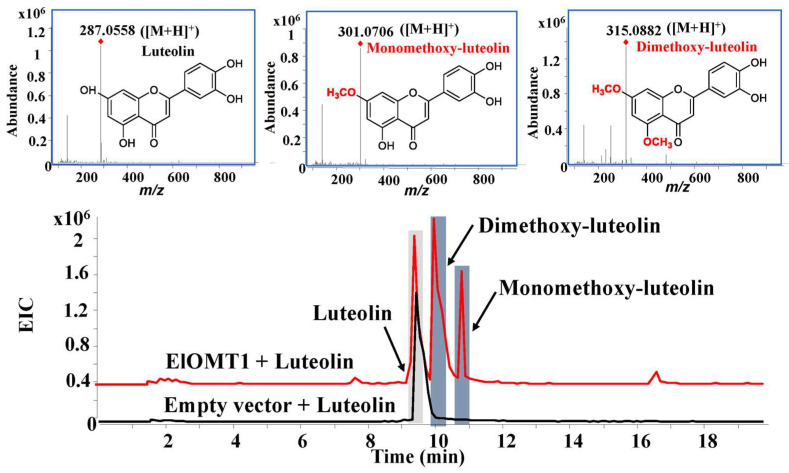
Results of luteolin substrate catalyzed by ElOMT1 in vitro. EIC, extract ion chromatogram.

The activities of recombinant protein ElOMT1 were investigated by using substrates, including caffeic acid, baicalein, naringenin, rutin, kaempferol, and luteolin in vitro. High-performance liquid chromatography–quadrupole time-of-flight mass spectrometry (HPLC-Q TOF-MS/MS) was used to analyze the products of the recombinant enzyme ElOMT1 and substrates. The results of recombinant enzyme catalysis in vitro showed that ElOMT1 was capable of catalyzing the methylation of caffeic acid, baicalein, and luteolin with broad regioselectivity. ElOMT1 with caffeic acid exhibited the monomethoxy-caffeic acid product *m*/*z* 195.0641 [M + H]^+^ and dimethoxy-caffeic acid product 209.0796 [M + H]^+^, along with a substrate peak at *m*/*z* 181.0493 ([M + H]^+^) (Figure 5). ElOMT1 with baicalein showed peaks at *m/z* 285.0764 ([M + H]^+^, monomethoxy-baicalein), *m*/*z* 299.0916 ([M + H]^+^, dimethoxy-baicalein), and *m*/*z* 335.0917 ([M + Na]^+^, trimethoxy-baicalein) (Figure 6). However, ElOMT1 exhibited regioselectivity with the substrate luteolin. ElOMT1 could catalyze two hydroxyl groups on the parent nucleus of the luteolin flavone to form methoxy groups but could not catalyze hydroxyl groups on the side chain (Figure 7).

Flavonoids are an important class of natural products with versatile applications, which are often used in food, dyes, and pharmaceutical fields [19]. Due to their extensive pharmacological activity, a variety of flavonoid drugs have been developed for the treatment of diseases [20]. In particular, flavonoids from natural sources have attracted increasing attention. However, flavonoids are mainly extracted from plants. Therefore, elucidating the flavonoid biosynthetic pathway is of great significance for enhancing flavonoid yield in plants. When plants receive abiotic stress factors, such as UV radiation, they will produce some secondary metabolites in response to this stress [21,22,23]. Flavonoids are important secondary metabolites in plants that respond to external abiotic stress [24,25].

When plants receive high doses of UV-B radiation, the plant leaves show severe wilting and curling. In addition, certain doses of UV-B radiation cause dwarfism in plants [26,27]. The damage caused by UV-B radiation to plants is mainly caused by the accumulation of reactive oxygen species (ROS), which harm cells by damaging proteins and DNA [28,29]. To counteract the damage caused by UV-B, plants increase their antioxidant enzyme activity, absorb UV-B, and scavenge ROS through flavonoid biosynthesis and accumulation [30]. However, there have been few studies on how plants can promote flavonoid biosynthesis after exposure to UV-B irradiation. In particular, the genes responding to the abiotic stress of UV-B in the biosynthetic pathway of flavonoids are still unclear.

In this study, we identified that a methyltransferase gene, *ElOMT1*, was involved in flavonoid biosynthesis, which could be significantly elevated via UV-B stimulation. The results indicated that *ElOMT1* might participate in the response to UV-B stimulation. Then, we cloned and expressed the *ElOMT1* gene and verified its activity in vitro. It was found that ElOMT1 could catalyze caffeic acid, baicalin, and luteolin to produce corresponding methylation products. Interestingly, ElOMT1 showed extensive catalysis of caffeic acid and baicalin. Indeed, we tested other flavonoid substrates, such as naringenin, rutin, and kaempferol, in ElOMT1, but none of them showed any activity. The results revealed that ElOMT1 exhibited differential catalytic activity on the flavonoid parent nucleus with regioselectivity. ElOMT1 catalysis has a substrate preference. When there are other groups outside the flavonoid parent nucleus, such as a sugar chain (rutin) or hydroxyl group (kaempferol), ElOMT1 causes substrate inactivation. Subsequently, we will consider screening more flavonoid substrates to expand the application range of the recombinant enzyme ElOMT1 in the future. The discovery of *ElOMT1* provides gene resources and target genes for studying the mechanism of UV-B irradiation of flavonoids in plants. We will further investigate the specific mechanism of the response to UV-B by overexpression or silencing the *ElOMT1* gene in concomitants. In addition, the discovery of *ElOMT1* provides an important gene for the production of methyl flavonoids through enzyme-catalyzed methods. Furthermore, it is worth noting that, in addition to functional gene methyltransferase responding to UV-B stimulation, some transcription factors also respond to the abiotic stress of UV-B. When analyzing the transcriptome data, we found that several transcription factors, such as HSF, WRKY, bHLH, and other transcription factor families, significantly increased or decreased after being stimulated via UV-B. It was suggested that these transcription factors might also be involved in the resistance to UV-B stimulation. Next, we will study these significantly changed transcription factors to further elucidate the mechanism of their response to UV-B.

## 3. Materials and Methods

### 3.1. Plant Materials and UV-B Treatments

*E. lathyris* sterilized seeds were grown on the medium of Murashige and Skoog to generate seedlings. For callus induction, 5 cm long sections of seedlings were excised and placed in a callus induction medium. After 3 weeks, the emerging callus tissues were separated from the upper stems (Figure S1) and cultured in the medium for UV-B treatments. UV-B radiation was generated using a TL 100W/01 tube in laboratory conditions under constant white light delivered through LEDs above the callus. An effective dose rate of ~D35 mW cm^−2^ irradiance at 311 nm normalized UV-B was provided for 3 h. Each group was tested with three biological replicates. The UV-B lamp was placed 45 cm above the callus. The control involved white light exposure for 3 h without UV-B [3]. Calluses were collected and immediately frozen in liquid nitrogen for RNA extraction after UV-B treatments.

### 3.2. RNA-Seq

The total RNA of each sample was extracted using the FastPure Universal Plant Total RNA Isolation Kit RC411 (Vazyme Biotech Co., Ltd., Nanjing, China), and RNA quality was tested with the Bioanalyzer 2100 system. Sequencing libraries were constructed using the RNA Library Prep Kit for Illumina according to the factory kit instructions. To obtain the appropriate size, the product was purified, and the size was selected for sequencing. The desired product was obtained through PCR and purified. Clustering of the index-coded samples was performed on a cBot Cluster Generation System [17]. After cluster generation, the library preparations were sequenced on the Illumina HiSeq X Ten platform at Wuhan Benagen Tech Solutions Company Limited (Wuhan, China). Raw reads were filtered by removing low-quality reads for the next analysis. The clean reads were applied for assembly through Trinity for each sample [31]. Tgicl was used for clustering assembly sequences and redundancy removal [32]. Assembly sequences that were no shorter than 200 bp were abstracted as genes for the next analysis. Values of fragments per kilobase of the transcript per million mapped fragments (FPKM) were used as a measure of gene expression levels. A *q* value < 0.05 and |log 2-fold change| > 1 were used as screening criteria for differentially expressed genes. Annotations were assigned to each unigene based on the top hit in BLASTX, which was used to search against the protein databases, with the nonredundant (Nr) protein database from GeneBank (http://www.ncbi.nlm.nih.gov, accessed on 16 January 2022) having the highest priority, followed by Swiss-Prot, KEGG, and eukaryotic KOG, in that priority order.

### 3.3. Recombinant Protein Expression in E. coli

The ORF of *ElOMT1* was obtained through PCR using cDNA as the template and cloned into pMAL-c4x (EcoR I/Sal I). The constructed plasmids were introduced into BL21 (DE3) for protein expression. The engineered strains of *E. coli* (harboring the recombinant plasmids) were cultured in 5 mL of Lysogeny broth (LB) liquid media supplemented with ampicillin antibiotics and incubated for 12 h at 37 °C. Then, 1 mL of the seed broth was cultured in 100 mL of LB at 37 °C for 3 h. Then, the engineered strains were induced using 0.2 mM IPTG, and they were incubated for an additional 18 h at 16 °C. The cells were collected after centrifugation (6000× *g*, 4 °C, 5 min) and resuspended in a binding buffer. The suspension was homogenized for 15 min (on ice) using a sonicator, which was set to a power of 30% and worked for 3 s with 5 s intervals. The cell debris was removed after centrifugation at 6000× *g* for 10 min under 4 °C. The supernatant was collected, and the recombinant enzyme was purified with an ÄKTA protein purification device. A 10% sodium dodecyl sulfate–polyacrylamide gel electrophoresis (SDS–PAGE) was used to detect the protein, and coloration was carried out with Coomassie brilliant blue. Assays of methylation activities were performed in 100 mM Tris-HCl (pH 7.5) with 0.5 mM of the substrate, 5 mM of *S*-adenosyl methionine, and 3 μL of the crude enzyme to obtain a reaction volume of 100 μL. Negative controls included the same reagents, with the exception being that the crude enzyme was boiled for 10 min. After incubating the mixtures for 12 h at 37 °C, the reaction was quenched using an equal volume of methanol, and ultrasound was conducted for 30 min. After centrifugation (12,000× *g*, 4 °C, 10 min), the suspension was used for the identification of the products through HPLC-Q TOF-MS/MS.

### 3.4. Product Analysis

A 1260 HPLC system coupled with a 6530-quadrupole time-of-flight mass spectrometer was applied for the analysis of bioconversion products from the recombinant enzyme. The chromatographic column was equipped with a ZORBAX C_18_ column. Mobile phase A consisted of a solution (0.1% formic acid) in water, whereas mobile phase B was composed of methanol. The gradient program was as follows: 20% B for 0–1 min; 20–80% B for 1–6 min; 80–100% B for 6–16 min; 100% B for 16–20 min; and 100–20% B for 20–25 min. The flow rate was set to 0.3 mL min^−1^, and the injection volume was 5 μL. The mass spectra were acquired in the positive ion mode with the acquisition parameters as follows: 4 KV capillary voltage; 10.0 L min^−1^ drying gas flow rate; 350 °C drying gas temperature; 120 V fragmentor voltage; and 35 psi nebulizer pressure. Mass spectra were recorded from 100 to 1500 *m*/*z*, and collision energies ranged from 10 to 60 eV. Compounds were identified through a comparison between molecular ions and fragmentation behaviors of reference standards or compounds identified in the literature. Relative quantification of compounds was performed via Agilent MassHunter Quantitative analysis (v_10.1) using the peak area based on extract ion chromatography.

## Data Availability

The datasets generated during and/or analyzed during the current study are available from the corresponding authors upon reasonable request. The transcriptome data have been uploaded to the NCBI (PRJNA1058867). The sequence of *ElOMT1* has been deposited in the GenBank with an accession number of OR902769.

## Supplementary Materials

The following supporting information can be downloaded at https://www.mdpi.com/article/10.3390/ijms25020782/s1.
