# Supplementary material for: Identification of One O-Methyltransferase Gene Involved in Methylated Flavonoid Biosynthesis Related to the UV-B Irradiation Response in Euphorbia lathyris
Source: Int J Mol Sci. 2024 Jan 8;25(2):782. doi: 10.3390/ijms25020782 (PMC10815478; doi:10.3390/ijms25020782)
Supplement: Supplementary file 1 [file ijms-25-00782-s001.zip › Supporting Information 1.pdf]

Supporting Information 1

Table S1 Statistics of output of the RNA-Seq

Sample	Raw reads	Raw bases	Clean reads	Clean bases	Q20_rate	Q30_rate	GC_ rate
ELC-UV0h-1	57,881,318	8,682,197,700	57,637,084	8,538,910,053	97.36%	92.52%	42.57%
ELC-UV0h-2	42,771,148	6,415,672,200	42,551,148	6,296,614,517	97.31%	92.48%	42.56%
ELC-UV0h-3	39,507,428	5,926,114,200	39,339,862	5,840,390,302	97.91%	93.64%	42.49%
ELC-UV3h-1	43,008,738	6,451,310,700	42,808,178	6,347,717,512	98.02%	93.96%	42.83%
ELC-UV3h-2	44,155,052	6,623,257,800	43,973,208	6,519,701,416	98.01%	93.89%	42.72%
ELC-UV3h-3	42,603,226	6,390,483,900	42,408,872	6,275,545,845	97.94%	93.75%	42.24%

Note: ELC-UV0h, the callus of *E. lathyris* was not exposed to UV-B irradiation; ELC-UV3h, the callus of *E. lathyris* were exposed to UV-B irradiation for 3h.

Table S2. GenBank accession numbers of MT proteins from other plants in Figure 3B

Genes	Species	Accession number
ElOMT1	*Euphorbia lathyris*	OR902769
BrF3'OMT	*Brassica rapa*	XP_009132451
ObFOMT1	*Ocimum basilicum*	AFU50295.1
AtOMT1	*Arabidopsis thaliana*	AAB96879.1
HvOMT1	*Hordeum vulgare*	ABQ58825.1
ChOMT	*Medicago sativa*	AAB48059.1
CrOMT2	*Catharanthus roseus*	Q8GSN1
ZmOMT1	*Zea mays*	ABQ58826.1
TaCOMT1	*Triticum aestivum*	Q84N28.1
HvF7OMT	*Hordeum vulgare*	CAA54616.1
CdFOMT5	*Citrus depressa*	BAU51794.1
PsOMT1	*Pinus sylvestris*	AQX17825.1
CaOMT2	*Citrus aurantium*	ADK97702.1
PfOMT3	*Perilla frutescens*	QOE76460.1
GmIOMT1	*Glycine max*	NP_001353843.1
IiOMT3	*Isatis tinctoria*	WET17731.1
PaCCoAOMT1	*Polypodiodes amoena*	QCY65250.1
ZvCCoAOMT	*Zinnia violacea*	AAA59389.1
GlSOMT9	*Glycine max*	NP_001236240.1
AnthOMT	*Solanum lycopersicon*	NP_001289828.1
EpCCoAOMT	*Euphorbia peplus*	WCJ17706.1
PaMTH1	*Podospora anserina*	4QVK_A
SbCCoAOMT	*Sorghum bicolor*	5KVA_A
ClOMT	*Curcuma longa*	UXX21556.1
IiOMT1	*Isatis tinctoria*	WET17729.1
IiOMT2	*Isatis tinctoria*	WET17730.1
AaCCoAOMT	*Artemisia annua*	PWA69367.1
HmOMT1	*Haplomitrium mnioides*	QCG81601.1
PaF6OMT	*Plagiochasma appendiculatum*	APX42106.1
MeOMT1	*Marchantia emarginata*	QCG81600.1
MpOMT	*Mucuna pruriens*	RDX82993.1

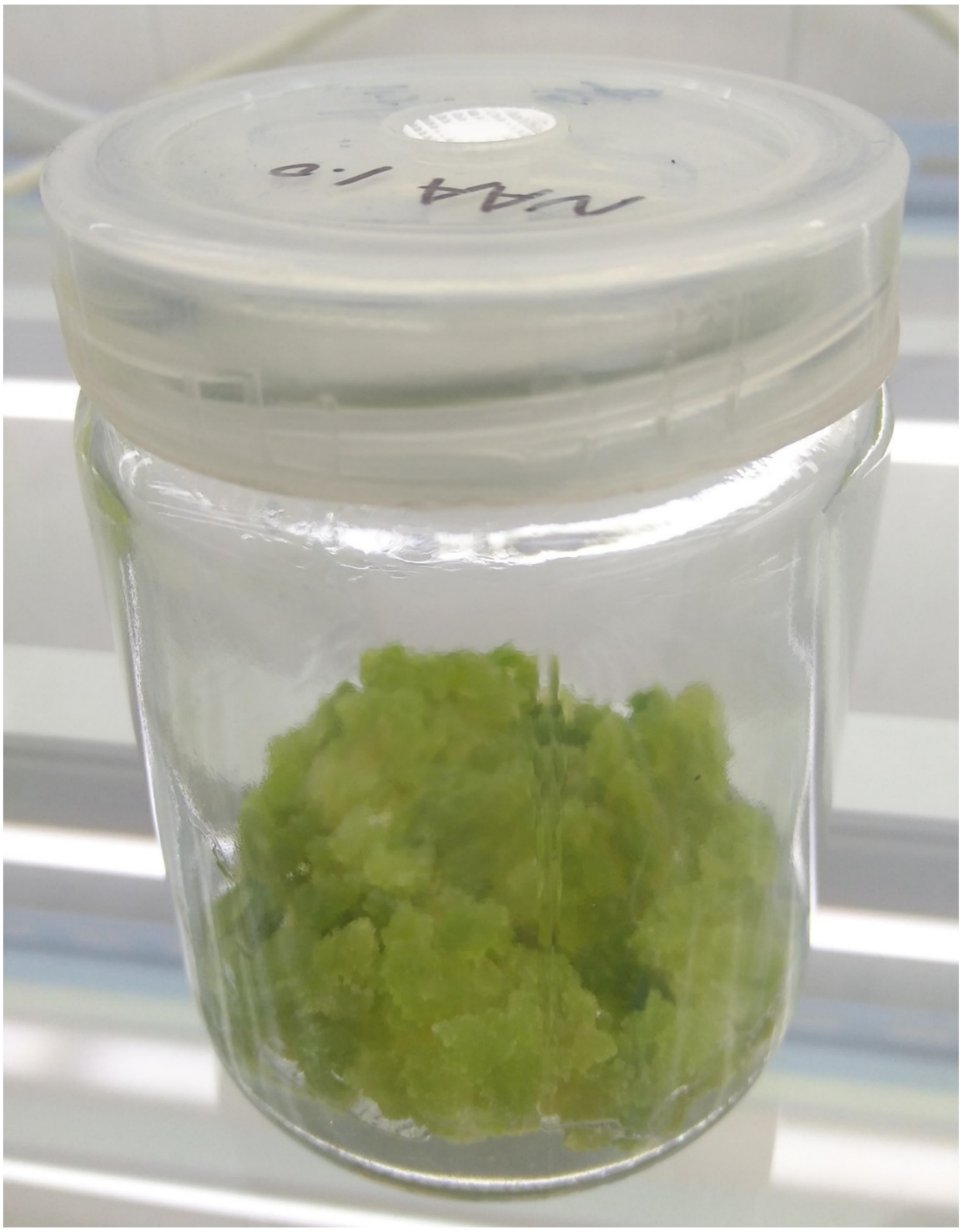

Figure S1 Picture of *E. lathyris* callus
